# Protein Network Analysis of the Serum and Their Functional Implication in Patients Subjected to Traumatic Brain Injury

**DOI:** 10.3389/fnins.2018.01049

**Published:** 2019-01-31

**Authors:** Xiu-Ying He, Qi-Qin Dan, Fang Wang, Yu-Kai Li, Song-Jun Fu, Nan Zhao, Ting-Hua Wang

**Affiliations:** ^1^Department of Anesthesiology, Institute of Neurological Disease, Translational Neuroscience Center, West China Hospital, Sichuan University, Chengdu, China; ^2^Institute of Neuroscience, Laboratory Zoology Department, Kunming Medical University, Kunming, China; ^3^Department of Neurosurgery, The First Hospital of Kunming, Kunming, China

**Keywords:** protein network analysis, the serum, ELISA, patients, traumatic brain injury

## Abstract

Traumatic brain injury (TBI) often leads to severe neurobehavioral impairment, but the underlying molecular mechanism remains to be elucidated. Here, we collected the sera from 23 patients (aged from 19 to 81 years old, third day after TBI as TBI-third group) subjected to TBI from The First Hospital of Kunming City, and the sera from 22 healthy donors (aged from 18 to 81 years old and as control group). Then, three samples from TBI-third group and three samples from control group were subjected to the protein microarray detection, and bioinformatics analysis. Then, enzyme-linked immunosorbent assay (ELISA) was used to verify significantly altered protein levels. Results showed that, when compared with the control group, all significantly differentially expressed proteins [DEPs, *P* < 0.05, FDR < 0.05, fold change (FC) > 2] contained 172 molecules in the TBI-third group, in which 65 proteins were upregulated, while 107 proteins were downregulated. The biological processes of these DEPs, mostly happened in the extracellular region and the extracellular region parts, are mainly involved in the regulation of cellular process, signaling and signal transduction, cell communication, response to stimuli, the immune system process and multicellular organismal development. Moreover, the essential molecular functions of them are cytokine activity, growth factor activity and morphogen activity. Additionally, the most significant pathways are enriched in cytokine–cytokine receptor interaction and PI3K-Akt signaling pathways among downregulated proteins, and pathways in cancer and cytokine–cytokine receptor interaction among upregulated proteins. Of these, we focused on the NGF, NT-3, IGF-2, HGF, NPY, CRP, MMP-9, and ICAM-2 with a high number of interactors in Protein–Protein Interaction (PPI) Network indicated by bioinformatics report. Furthermore, using ELISA test, we confirmed that all increase in the levels of NGF, NT-3, IGF-2, HGF, NPY, CRP, MMP-9, and ICAM-2 in the serum from TBI patients. Together, we determined the screened protein expressional profiles in serum for TBI patients, in which the cross-network between inflammatory factors and growth factors may play a crucial role in TBI damage and repair. Our findings could contribute to indication for the diagnosis and treatment of TBI in future translational medicine and clinical practice.

## Introduction

Traumatic brain injury (TBI) refers to an intracranial injury when external forces act on the brain and bring about severe neurobehavioral impairment (Maas et al., 2008). In recent years, the incidence, mortality and disability of TBI have been on the rise (Bruns and Jagoda, 2009; Mondello et al., 2014). TBI has become a major cause of death and disability worldwide, especially in children and young people (Alves and Bullock, 2001; Hannay et al., 2004). The degree of brain damage in TBI is divided into mild, moderate and severe categories (Saatman et al., 2008). According to the severity, patients will present different physical, cognitive, social, emotional, and behavioral symptoms (Kushner, 1998; Whigham et al., 2011) and have outcomes from full recovery to permanent disability or death (Frey, 2003; Crooks et al., 2007; Brown et al., 2008; Maas et al., 2008; Nicholl and Lafrance, 2009). The current treatment measures are nothing more than drug treatment, emergency surgery or surgery after a few years (Maas et al., 2008; Kochanek and Tasker, 2009). With the development of medicine, although the current treatment measures have been greatly improved, they are not satisfactory. Consequently, further providing the molecular basis of TBI for clinical treatment has become inevitable.

Traumatic brain injury includes primary and secondary brain damage. Primary brain injury occurs during the initial period of injury due to displacement of the physical structure of the brain (Scalea, 2005). Secondary brain injury, which may be due to primary injury or independent of its cause (Porth and Gaspard, 2014), occurs gradually and involves a series of cellular processes including damage to blood–brain barrier, release of inflammatory factors, free radical overload, excessive release of neurotransmitter glutamate (excitotoxicity), influx of calcium and sodium ions, and mitochondrial dysfunction (Xiong et al., 1999; Park et al., 2008). Nowadays, some molecules have been found to participate in the development of secondary brain injury. The gene expression profile in the rat cerebral cortex with the secondary injury of TBI was analyzed by bioinformatics analysis and the expression change of the genes was found after 48 h of TBI, whose molecular functions were steroid biosynthesis, cell cycle, metal ion transport, inflammation and the apoptosis function (Ou et al., 2014). In addition, by using microarrays and bioinformatics analysis, Puhakka et al. (2017) confirmed 3 months after TBI miR-139-5p was downregulated and the NOTCH1 interactome was upregulated. Then, by genome-wide approach, Lamprecht et al. (2016) found that after TBI, sorting protein-related receptor with A-type repeats (SORLA) may be involved in the post-traumatic cascade linking TBI to Alzheimer’s disease. Moreover, the complement factor 9 (C9), complement factor B (CFB) and aldolase c (ALDOC) in the serum of rat TBI models rose in the early stage of injury, while hypoxia inducing factor (HIF) 1α, amyloid precursor protein (APP) and Williams-Beuren syndrome chromosome region 17 (WBSCR17) increased at the late stage of injury (Thelin et al., 2016). Furthermore, what proteins have changed in the serum of human with TBI? Bogoslovsky et al. (2016) enrolled 34 TBI subjects and 69 healthy volunteers and found an increase in glial fibrillary acidic protein (GFAP), microtubule-associated protein tau and amyloid β peptide (Aβ42) at days 0, 30, and 90 after TBI. In summary, there have been great changes in the serum proteins after TBI, but there are still few studies in this field, especially regarding the changes in human serum protein.

As the secondary injury gradually occurs and is more prominent to the body, the patient’s condition may gradually deteriorate (Sullivan et al., 2000; Reinert and Bullock, 2002). Of the various factors, the role of inflammatory factors cannot be ignored. Previous study has reported that the gene expression in the ipsilateral hippocampus of mice with mild TBI was changed, which mapped to many ontologies and molecular pathways related to inflammation and neurological physiology/pathology, including neurodegenerative disease (Tweedie et al., 2016). Simultaneously, Nwachuku et al. (2016) found that CSF concentrations of inflammatory biomarkers (IL-1β, IL-6, TNF-α, IFN-γ, IL-12p70, IL-10, and IL-8) had a significant association with 6-month neurological outcome, with the favorable outcome group having lower concentrations of these biomarkers on average, in comparison to the poor neurological outcome group after severe TBI.

Growth factor is a protein, or a steroid hormone, capable of stimulating cellular growth, proliferation, and differentiation (Hauer et al., 2009). At present, the role of growth factors in TBI have also been revealed. The expression of BDNF and its receptors at the acute phase following penetrating TBI of rat has been reported firstly to be altered (Rostami et al., 2014). Additionally, in the blast-induced traumatic brain injury of swine, vascular endothelial growth factor (VEGF) was discovered to increase (Ahmed et al., 2012). Moreover, the mRNA expression of components and targets of the TGF-β, BMP, and activin signaling pathways in the subventricular zone (SVZ) and the hippocampal dentate gyrus (DG) was significantly altered after cortical injury in mice, which then induced expression of Runt-related transcription factor-1 (Runx1) that can interact with intracellular TGF-β Smad signaling pathways and promotes microglial cell activation and proliferation and neural stem cell (NSC) proliferation after TBI (Logan et al., 2013).

Although some molecules have been found to be involved in repair and deterioration of the injury, a series of cellular processes are involved after TBI, especially in the development of secondary brain injury. Therefore, these processes cannot be the result of the action of one or more molecules. Accordingly, it is necessary to continue to explore the key players after TBI injury. In this study, serum protein microarray technology and bioinformatics analysis were used to investigate what molecules in the serum of TBI patients make a difference to these changes, and what functions and pathways these molecules are mainly enriched in. In particular, the expression of growth factors and inflammatory factors in serum of TBI patients was confirmed.

## Materials and Methods

### Ethics Statement and Grouping

Human serum samples used in this study were provided by The First Hospital of Kunming. The patients did not receive special treatment including radiotherapy and chemotherapy. The age of the patients ranged from 18 to 81 years old (mean 46.4 years old). These 23 serum samples were collected third day after TBI and served as TBI-third group. Moreover, the sera from 22 healthy volunteers without cerebral injury and other organic diseases were selected for the control group. Patients’ data was extracted by two independent reviewers, and the study type of the dataset was protein expression profile studies. Any discrepancies between reviewers were resolved by consensus or a third reviewer. This study was carried out in accordance with the recommendations of the Declaration of Helsinki, the Ethical Committee of The First Hospital of Kunming (reference number 2014-2). The protocol was approved by the Ethical Committee of The First Hospital of Kunming. All subjects gave written informed consent in accordance with the Declaration of Helsinki.

### Collection of Human Serum Samples

Grouping information and all the patients’ information is shown in Tables 1, 2, respectively. The whole blood of the patients was collected with coagulant tubes. After blood collection, the coagulant tubes (SANLI, Jiangsu, China) were gently reversed, and the blood was mixed four to five times and then stood upright at room temperature until the blood was completely solidified (usually about 1 h). Within 2 h after blood collection, all the serum samples were retrieved and transferred to the laboratory of the Institute of Neuroscience, Kunming Medical University. After the filtering process according to the standards, the coagulant tubes of 23 human serum samples on the TBI-third group were centrifugated with 1,000 × *g* for 10 min, and the serum was isolated. The collected serum was transferred to 1.5 ml microtubes (Axygen, United States) and divided into 200–300 μl/tube. All samples were immediately frozen in liquid nitrogen, and then stored at -80°C. Moreover, the serum obtained from control group underwent the same procedures. Finally, the samples were filled in a vessel with dry ice and transported to Shanghai Kangcheng Biological Engineering Co., Ltd. for further study.

**Table 1:** Patients’ grouping and test in both groups.

Group	Protein microarray	ELISA/blood biochemical examination
Control group	3	19
TBI-third group	3	20


**Table 2:** Baseline clinic data of patients.

Items	Sub items/unit	Control group	TBI-third group
Gender	Male	53%	83%
	Female	47%	17%
Age	Years old	44.4 ± 20.56	46.4 ± 16.24
Hematoma	Intracerebral	0	45%
	Epidural	0	30%
	Subdural	0	10%
	No	100%	15%
Injured part	Forehead	0	70%
	Partes temporalis	0	45%
	Facies parietalis	0	35%
	Occiput	0	15%
Treatment	Surgery	*NO*	30%
	Expectant treatment	*NO*	70%
GCS	Scores	15 ± 0	13.25 ± 0.39

**Other Indexes**	**Normal reference range**	**Mean ±** **SD**	**Mean ± SD**

HGB	110–160 g/L	-	126.73 ± 16.21
WBC	(4–10) × 10^9^/L	-	9.06 ± 4.48
NEUT	50–70%	-	74.53 ± 9.27
LYM	20–40%	-	18.95 ± 7.87
MONO	3–8%	-	5.55 ± 2.48
PLT	(100–300) × 10^9^/L	-	165.33 ± 29.48
FIB	2.0–4.0 g/L	-	3.17 ± 1.31
APTT	31–43 s	-	34.16 ± 3.95
PT	11–13 s	-	13.20 ± 1.00
INR	0.94–1.29	-	1.01 ± 0.10
CREA	53–115 μmol/L	64.75 ± 10.39	80.14 ± 18.89**
BUN	2.8–7.6 mmol/L	5.46 ± 1.54	4.18 ± 1.45*
UA	208.3–428.4 μmol/L	377.06 ± 116.07	196.13 ± 74.24***
TP	66–83 g/L	73.69 ± 3.21	67.27 ± 6.55***
ALB	35–52 g/L	44.17 ± 3.19	36.22 ± 5.00***
GLB	20–30 g/L	29.52 ± 2.77	31.06 ± 3.98
A/G	1.10–2.50	1.51 ± 0.22	1.22 ± 0.27***
AST	<50 U/L	24.54 ± 7.42	30.02 ± 14.58
ALT	<50 U/L	17.56 ± 13.11	31.48 ± 13.17**
ALP	30–120 U/L	72.97 ± 22.97	78.51 ± 34.78
GGT	8–57 U/L	21.66 ± 14.19	45.17 ± 24.31***
TBIL	5–21 μmol/L	12.39 ± 5.68	13.20 ± 6.05
DBIL	<3.4 μmol/L	2.51 ± 1.35	2.97 ± 1.51
IBIL	2.0–17.0 μmol/L	9.88 ± 4.36	10.23 ± 4.70
TG	0.4–1.7 mmol/L	2.46 ± 1.32	1.42 ± 0.83
TC	3–5.7 mmol/L	4.28 ± 0.81	4.04 ± 1.40
LDL-C	1.89–4.21 mmol/L	2.33 ± 0.66	1.98 ± 0.58
HDL-C	1.03–1.55 mmol/L	0.80 ± 0.22	1.09 ± 0.41


*^∗^TBI-third group vs control group, *P* < 0.05, ^∗∗^TBI-third group vs control group, *P* < 0.01, ^∗∗∗^TBI-third group vs control group, *P* < 0.001.*

### Protein Microarray Analysis

To detect the DEPs, three samples in TBI-third group and three samples in the control group (these six patients’ basic information is shown in Supplementary Data Sheet S1) were performed the protein microarray analysis. Firstly, the serum samples were diluted with PH = 8.0 1 × PBS five times, and were dialyzed. Then the protein concentration of the serum samples was measured according to the operating instructions of the KC^TM^ BCA protein quantification kit (KangChen Bio-tech, Shanghai, China). Next, the samples and 1× labeling reagent were added into new tubes, mixed, and incubated for 30 min at room temperature. After that, stop solution was used for stopping the label reaction and the biotin labeled samples were dialyzed three times with dialysis tubes. Meanwhile, the RayBio^®^ Biotin label-based human antibody array 1 and 2 (including 1,000 antibodies: cytokines, chemokines, adipokines, growth factors, proteases, soluble antibodies, adhesion factors and other proteins, which all are shown in Supplementary Data Sheet S2) were balanced for 1 h at room temperature and placed at the vacuum dryer for 1 h. Then 400 ul 1× blocking buffer was added into each chip well to incubate on a shaker for 1 h at room temperature. After discarding the blocking buffer, 400 μl of the sample was added to each well and incubated overnight at 4°C with shaking. When the sample was removed, 1× wash buffer I and II were used for washing the arrays 5 min/time for three times, respectively. After that, 400 ul 1× Cy3 equivalent (diluted by 1× blocking buffer) was added into each well and incubated for 2 h at room temperature with shaking and protection from light. Similarly, the arrays were washed with 1× wash buffer I and II. Finally, the InnoScan 300 Microarray Scanner (Parc d’Activités Activestre; Carbonne-France) with wave length 532 nm and resolution 10 μm was used for detecting the fluorescent signal on the arrays. The RayBio^®^ Analysis Tool software and data analysis software of AAH-BLG-1000 were used to extract and analyze the data. The obtained fluorescence intensity data first subtracted the background and was normalized to the Positive Control signals. Differentially expressed proteins (DEPs) were obtained by comparison of the signal values between groups based on *P* < 0.05 by *t*-test, FDR < 0.05, and fold change (FC) > 2 or <0.5. In TBI-third group, the proteins more than twice the relative expression of that in the control group were defined as up-regulated proteins, and the proteins that were less than 0.5 times the relative expression of that in the control group were defined as down-regulated proteins.

### Gene Ontology and KEGG Pathway Analysis of DEPs

In this study, we applied Gene Ontology (GO) analysis and Kyoto Encyclopedia of Genes and Genomes (KEGG) Pathway to analyze the DEPs by using String online tools^1^. GO analysis was employed to annotate genes and gene products including molecular function, biological process, and cellular component. KEGG is a knowledge base for systematic analysis of gene functions, comprising a series of genome and enzymatic approaches and genomic information with higher order functional information (Kanehisa and Goto, 2000). Accordingly, it was used for systematic analysis of gene function and related high-level genome functional information of DEPs.

### Integration of Protein–Protein Interaction (PPI) Network Analysis

STRING version 10.0 covers 9,643,763 proteins obtained from 2,031 organisms (Szklarczyk et al., 2015). The STRING database (see footnote 1) is utilized to assess and predict the PPIs comprising direct (physical) and indirect (functional) associations. In order to assess the interactional relationships and build a PPI network among the DEPs, STRING tool was employed and established a PPI network according to the function and pathway enrichment analysis. *P* < 0.05 was considered statistically significant.

### Automatic Blood Biochemical Examination

The Beckman Coulter Chemistry Analyzer (AU480) (Beckman Coulter K.K., Tokyo, Japan) was used to detect the serum level of the indicators that reflect the liver and kidney function and lipid status. The Lis 2.2 software (Shanghai Medical InfoTech Co., Ltd., China) was operated for result display. Reagent detection, ion calibration, and quality control must be performed every time the sample is tested. Therefore, these three steps were performed first. Firstly, the standard reagents (Beckman Coulter Laboratory Systems Co., Ltd., Suzhou, China) were placed in the corresponding positions and the sample cups (Kangjie Medical Devices Co., Ltd., Jiangsu, China) filled with W1 cleaning solution and W2 cleaning solution (Beckman Coulter, Inc., Brea, CA, United States) were laid in the stat table. Then the reagent check was started. After, ISE High Serum Standard and ISE Low Serum Standard (Beckman Coulter, Inc., Brea, CA, United States) were then placed in the S-H and S-L positions on the stat table, respectively, for all ion calibrations. Subsequently, the quality control test was conducted. Quality control is the detection of the stability of the reagents. The sample cups with Control Serum 1 and Control Serum 2 (Beckman Coulter Ireland Inc., Co., Clare, Ireland) were placed on the sample rack (Beckman Coulter K.K., Tokyo, Japan) for quality control tests. After the tests were completed, the result was transmitted to the Lis 2.2 software. When all the tests were passed, blood biochemical examination of the samples could be performed. Clean sample cups with 300 μl of the serum sample were put on the sample racks in turn, and the items to be detected were selected for sample examination. The results were also displayed on the Lis 2.2 software.

### Enzyme-Linked Immunosorbent Assay (ELISA)

In order to determine the levels of human NGF, NT-3, HGF, IGF-2, NPY, CRP, ICAM-2, and MMP-9 in the patients’ serum, the enzyme-linked immunosorbent assay (ELISA) kits were purchased from MEIMIAN (Kete Biological Technology Co., Ltd., Jiangsu, China) and used according to the manufacturer’s instructions. Firstly, the microtiter plates were coated with purified NGF, NT-3, HGF, IGF-2, NPY, CRP, ICAM-2, and MMP-9 antibodies, respectively. Then blank wells, the standard wells and sample wells were set. In the sample wells, 25 μl of the sample diluent and 25 μl of a sample to be tested were added to the bottom of the wells (the final dilution of the sample is two times). At the same time, 50 ul of the standards of known concentration were added into the standards wells. Nothing was added to the blank well. After that, 100 μl HRP-conjugated reagent was added to each well except the blank well, and the plates were covered with adhesive films, mixed gently and incubated for 60 min at 37°C in the incubator (Thermo, Marietta, OH, United States). Next, the plates were rinsed five times with 1× washing solution and patted dry. Then 50 μl of substrate A and 50 μl of substrate B were added to each well and gently mixed. After incubating in the dark at 37°C for 15 min, the reaction was stopped by 50 μl of stop solution (blue at this time turned yellow). Within 15 min, the absorbance (optical density value) of each well was measured with a spectrophotometer (Thermo Fisher Scientific, Vantaa, Finland) at a wavelength of 450 nm. Finally, the linear regression equations of the standard curves were calculated using the concentration and the OD value of the standards and then the concentration of NGF, NT-3, HGF, IGF-2, NPY, CRP, ICAM-2, and MMP-9 in the serum was calculated.

### Statistical Analysis

The measurement data were statistically analyzed with an independent sample *t*-test using SPSS 16.0 software (IBM Corporation, Armonk, NY, United States). *P* < 0.05 was considered as statistical significance. The concentrations of NGF, NT-3, HGF, IGF-2, NPY, CRP, ICAM-2, and MMP-9 in the serum were shown by mean ± standard error (SEM) and 95% confidence interval (CI). In addition to the concentrations of these molecules, other measurement data were represented as mean ± standard deviation (SD). The enumeration data was also analyzed by SPSS 16.0, and represented with the percentage.

## Results

### Baseline Data of the Patients

Baseline characteristics of all patients are shown in Table 2. The patients in the control group do not suffer from cerebral injury or another organic disease. In addition to traumatic brain damage, the patients of TBI-third group also aren’t afflicted with other diseases. The percentage of male and female who were embedded in the control group was 53% and 47%, respectively, but 85% and 15% in TBI-third group, respectively. The ratio of male and female included in the two groups was not consistent. However, the mean age of patients in the control group and TBI-third group were 44.4 ± 20.56 and 46.4 ± 16.24 years old, respectively, with no statistical difference (*P* = 0.733). Besides, the difference in creatinine (CREA), urea nitrogen (BUN), and uric acid (UA) between the two groups was statistically significant (*P* < 0.05), but they were all within the normal reference range. Therefore, the kidney function was not considered to be impaired in both groups. At the same time, the total protein (TP), albumin (ALB), globulin (GLB), albumin/globulin ratio (A/G), alanine aminotransferase (ALT), aspartate aminotransferase (AST), alkaline phosphatase (ALP), gamma-glutamyltranspeptidase (GGT), total bilirubin (TBIL), direct bilirubin (DBIL) and indirect bilirubin (IBIL), which all reflected the liver function, in the serum of control group and TBI-third group were also completely within the normal reference range, indicating good liver function in both groups. Moreover, the triglyceride, total cholesterol (TC), low density lipoprotein cholesterol (LDL-C) and high-density lipoprotein cholesterol (HDL-C), which reflected the state of blood lipid, did not differ between the two groups (*P* > 0.05). In addition to HDL-C that was less than the lower limit of the normal reference range in control group, TG, TC and LDL-C in control group, and TG, TC, LDL-C, and HDL-C in TBI-third group were all within the normal reference range, suggesting that the blood lipid level in both groups were almost normal. Furthermore, the Glasgow Coma Scale (GCS) scores of patients in control group and TBI-third groups were 15 ± 0 points and 13.25 ± 0.39 points, respectively, and the difference was statistically significant (*P* = 0.000). Additionally, CT examination revealed that the TBI patients had lesions in the forehead (70%), partes temporalis (45%), facies parietalis (35%), and occiput (15%). Hematoma occurred in 85% of the patients’ lesions, which appeared in intracerebral (45%), epidural (30%), and subdural (10%). For the treatment of hematoma, 70% of patients were treated conservatively, and only 30% of patients were treated by surgery to relieve hematoma compression. Blood examination and coagulative function evaluation showed that platelet (PLT), fibrinogen (FIB), activated partial thrombin time (APTT), prothrombin time (PT), and international normalized ratio (INR) of TBI patients were within the normal reference range, illustrating that the TBI patients had normal coagulation function and no possibility of spontaneous bleeding, that meant the hematomas were caused by trauma. Simultaneously, blood examination showed that the hemoglobin (HGB) in TBI patients was 126.73 ± 16.21 g/L, demonstrating that there was no massive hemorrhage after TBI. What’s more, the white blood cells (WBCs) were (9.06 ± 4.48) × 10^9^/L, and the proportions of neutrophil granulocyte (NEUT), lymphocyte (LYM), and monocyte (MONO) were (74.53 ± 9.27)%, (18.95 ± 7.87)%, and (5.55 ± 2.48)%, respectively. It can be seen that WBC and NEUT were elevated, while LYM and MONO were within normal reference range, revealing that there existed acute inflammatory response in TBI-third patients.

### Heat Map Analysis of DEPs

**FIGURE 1:**
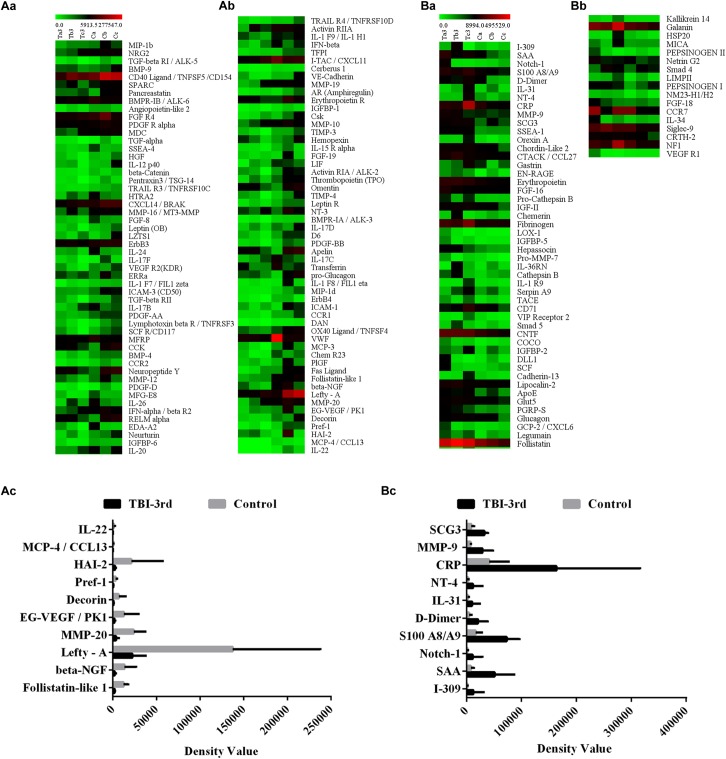
The expression profile of all the differentially expressed proteins (DEPs) in TBI-third group and control group. Here the protein expression was detected by protein microarray. In the heatmap, each column represents a sample, and each row represents a single protein. **(A-a,A-b)** Represent the down-regulated proteins, and the **(B-a,B-b)** represent the up-regulated proteins. **(A-c,B-c)** Show the top 10 of the lowest fold change proteins and the top 10 of the highest fold change proteins, respectively. The color scale shows the relative expression level of proteins in certain slide: The colors from green (left) to red (right) indicate the low to high relative expression levels, in which the green at the far left is the lowest relative expression level and the red at the far right is the highest relative expression level.

The expression levels of all significantly DEPs were shown in the heat map, which comprised 107 downregulated proteins and 65 upregulated proteins (Figure 1A-a,b,B-a,b and Supplementary Data Sheet S3). Moreover, the top 10 of downregulated proteins are Follistatin-like 1, beta-NGF, Lefty-A, MMP-20, EG-VEGF/PK1, Decorin, Pref-1, HAI-2, MCP-4/CCL13 and IL-22 (Figure 1A-c and Supplementary Data Sheet S3), and the top 10 of upregulated proteins are I-309, SAA, Notch-1, S100 A8/A9, D-Dimer, IL-31, NT-4, CRP, MMP-9 and SCG3 (Figure 1B-c and Supplementary Data Sheet S3).

### GO Function Analysis of Differential Expressed Proteins

The GO is a set of dynamic controlled vocabulary and used to describe the role of gene and protein with three major categories of information including biological process, cellular component, and molecular function. Biological process mainly involves a biological objective to which the gene or gene product contributes, and this process may be accomplished by one or more ordered assemblies of molecular functions. In this study, the DEPs were analyzed in relevant biological processes by gene annotations. The results indicated that the biological processes of downregulated proteins were involved in 212 categories, and the top 10 of biological processes were shown in Table 3A and Supplementary Data Sheet S4. Moreover, the biological processes of upregulated proteins were mainly 115 categories, and the top 10 biological processes were screened and shown in Table 3A and Supplementary Data Sheet S5. Among the three categories of GO, cellular component is related to the activation of a gene product. In this study, we found that the most expression of downregulated proteins and upregulated proteins both happened in the extracellular region, extracellular region part and extracellular space (Table 3B and Supplementary Data Sheets S4, S5). Molecular function is characterized by the biochemical activity of a gene product consisting of specific binding to ligands or structures. The molecular functions of downregulated proteins were mainly involved in receptor binding, protein binding, cytokine activity, growth factor activity, chemoattractant activity, cytokine receptor binding, C-C chemokine receptor activity cytokine binding, morphogen activity, and BMP binding (Table 3C and Supplementary Data Sheet S4), while the molecular function of upregulated proteins were consisting of protein binding, receptor binding, endopeptidase activity, growth factor activity, lipoprotein particle binding, and interleukin-6 receptor binding (Table 3C and Supplementary Data Sheet S5).

**Table 3A:** The top 10 biological process based on protein count of differentially expressed proteins (DEPs).

DEPs	Item description	Protein count and matching proteins
Downregulated DEPs	Single-organism cellular process	38: ADAM11, APLN, CCL15, CCL7, CCR1, CCR2, CDH5, CER1, CHGA, CTNNB1, DLK1, EDA, ERBB3, ESRRA, FASLG, FGF8, FSTL1, HGF, ICAM2, IFNB1, IL17B, IL17C, IL24, IL26, LIF, LZTS1, MFGE8, MMP20, MPL, NBL1, NGF, NPY, NRG2, NRTN, NTF3, PGF, RTTN, VWF
	Single organism signaling	33: ADAM11, APLN, CCL15, CCL7, CCR1, CCR2, CDH5, CER1, CSK, CTNNB1, DLK1, EDA, ERBB3, ESRRA, FASLG, FSTL1, HGF, HTRA2, ICAM2, IFNB1, IL17B, IL17C, IL26, LIF, MPL, NGF, NPY, NRG2, NRTN, NTF3, PGF, SPARC, TF
	Cell communication	33: ADAM11, APLN, CCL15, CCL7, CCR1, CCR2, CDH5, CER1, CSK, CTNNB1, DLK1, EDA, ERBB3, ESRRA, FASLG, FSTL1, HGF, HTRA2, ICAM2, IFNB1, IL17B, IL17C, IL26, LIF, MPL, NGF, NPY, NRG2, NRTN, NTF3, PGF, SPARC, TF
	Response to stimulus	32: ADAM11, APLN, CCL15, CCR1, CCR2, CDH5, CER1, CHGA, CTNNB1, DCN, DLK1, ERBB3, ESRRA, FASLG, HGF, ICAM2, IFNB1, IL17C, IL17D, IL17F, IL26, ITLN1, LIF, MFGE8, MPL, NGF, NPY, NRG2, NRTN, NTF3, TFPI, VWF
	Multicellular organismal development	31: ANGPTL2, APLN, CCBP2, CCK, CDH5, CER1, CSK, CSRP2, CTNNB1, EDA, ERBB3, ERBB4, ESRRA, FASLG, FGF8, HTRA2, IFNB1, IL17F, LZTS1, MFGE8, MFRP, MMP20, NBL1, NPY, NRG2, PGF, RTTN, SPARC, SPINT2, TF, TIMP2
	Signal transduction	31: ADAM11, APLN, CCL15, CCL7, CCR1, CCR2, CDH5, CER1, CSK, CTNNB1, DLK1, EDA, ERBB3, ESRRA, FASLG, FSTL1, HGF, HTRA2, ICAM2, IFNB1, IL17C, LIF, MPL, NGF, NPY, NRG2, NRTN, NTF3, PGF, SPARC, TF
	Cellular response to stimulus	31: ADAM11, APLN, CCL15, CCR1, CCR2, CDH5, CER1, CHGA, CTNNB1, DLK1, EDA, ERBB3, ESRRA, FASLG, FSTL1, HGF, HTRA2, ICAM2, IFNB1, IL17C, IL24, LIF, MPL, NGF, NPY, NRG2, NRTN, NTF3, SPARC, TF, TIMP2
	Single-multicellular organism process	30: ANGPTL2, CCBP2, CCR2, CDH5, CER1, CHGA, CSRP2, CTNNB1, EDA, ERBB3, ERBB4, ESRRA, FASLG, FGF8, HTRA2, IFNB1, IL17F, LZTS1, MFGE8, MFRP, MPL, NBL1, NPY, NRG2, PGF, RTTN, SPINT2, TFPI, TIMP2, VWF
	Regulation of metabolic process	30: APLN, CCK, CCR1, CCR2, CER1, CSK, CTNNB1, DCN, DLK1, EDA, ERBB3, ERBB4, ESRRA, FASLG, FGF8, FSTL1, HGF, HPX, HTRA2, IFNB1, IL24, IL26, ITLN1, LIF, LZTS1, NGF, NTF3, SPINT2, TF, TFPI
	Positive regulation of biological process	29: APLN, CCK, CCL7, CCR1, CCR2, CHGA, CTNNB1, DCN, ERBB3, ERBB4, ESRRA, FASLG, HGF, HPX, HTRA2, ICAM2, IFNB1, IL17B, IL17F, IL26, ITLN1, LIF, MFGE8, NBL1, NPY, NTF3, SPARC, TF, TIMP2
Upregulated DEPs	Positive regulation of biological process	26: ADAM17, APOE, CCR7, CDH13, CNTF, CRP, CTSB, DLL1, EPO, FST, HCRT, IGF2, IL34, ITGA2B, KITLG, KLK14, LGMN, MFNG, MMP9, PGLYRP1, RARRES2, SAA1, SMAD4, SMAD5, TFRC, VIPR2
	Multicellular organismal process	24: ADAM17, APOE, CCR7, CDH13, CHRDL2, CNTF, CTSB, DAND5, DLL1, FGF18, FST, GAL, KITLG, KLK14, LGMN, MFNG, NTNG2, OLR1, PAPPA, PGA5, RARRES2, SAA1, SCG3, TFRC
	Regulation of macromolecule metabolic process	23: ADAM17, APOE, CCR7, CDH13, CNTF, CRP, DLL1, EPO, FGF18, FST, GAL, GCG, HCRT, IGF2, IL34, KITLG, MMP9, NF1, RARRES2, SAA1, SERPINA9, SMAD4, SMAD5
	Signal transduction	22: ADAM17, APOE, CCL1, CCR7, CDH13, CNTF, CTSB, DLL1, EPO, FGF18, FST, GAL, GCG, HCRT, IGF2, ITGA2B, LGMN, MMP9, PGLYRP1, SIGLEC9, SMAD4, SMAD5
	Positive regulation of cellular metabolic process	21: ADAM17, APOE, CCR7, CDH13, CNTF, CRP, DLL1, EPO, FGF18, GAL, GCG, IGF2, IL34, KITLG, MMP9, NF1, RARRES2, SAA1, SMAD4, SMAD5, VIPR2
	Negative regulation of cellular process	21: ADAM17, APOE, CCR7, CDH13, CHRDL2, CNTF, CRP, CTSB, DAND5, EPO, FST, HCRT, KITLG, KLK14, LGMN, MMP9, NTF4, PGLYRP1, SERPINA9, SMAD4, VIPR2
	Negative regulation of biological process	21: ADAM17, APOE, CCR7, CDH13, CHRDL2, CNTF, CRP, CTSB, DAND5, EPO, FST, HCRT, KITLG, KLK14, MMP9, NTF4, PGLYRP1, SAA1, SERPINA9, SMAD4, VIPR2
	Positive regulation of cellular process	21: ADAM17, APOE, CCR7, CDH13, CNTF, CRP, DLL1, EPO, HCRT, IGF2, IL34, ITGA2B, KITLG, KLK14, MFNG, MMP9, RARRES2, SAA1, SMAD4, SMAD5, VIPR2
	Regulation of signaling	20: ADAM17, APOE, CCR7, CDH13, CHRDL2, CNTF, DAND5, DLL1, EPO, FST, GAL, HCRT, IGF2, KITLG, KLK14, LGMN, MFNG, MMP9, SAA1, SMAD4
	Regulation of cell communication	20: ADAM17, APOE, CCR7, CDH13, CHRDL2, CNTF, DAND5, DLL1, EPO, FST, GAL, HCRT, IGF2, KITLG, KLK14, LGMN, MFNG, MMP9, SAA1, SMAD4


**Table 3B:** Cellular component of differentially expressed proteins (DEPs).

DEPs	Item description	Protein count and matching proteins
Downregulated DEPs	Extracellular region	40: ANGPTL2, APLN, CCL15, CCL7, CER1, CHGA, CSK, CTNNB1, DCN, DLK1, EDA, ERBB3, ERBB4, ESRRA, FASLG, FGF8, FSTL1, HPX, ICAM2, IFNB1, IL17B, IL17C, IL17D, IL17F, IL24, IL26, ITLN1, LIF, MFGE8, MMP20, NBL1, NGF, NPY, NRG2, NRTN, NTF3, SPINT2, TF, TFPI, VWF
	Extracellular region part	37: ANGPTL2, APLN, CCK, CCL15, CCL7, CER1, CSK, CTNNB1, DCN, DLK1, ERBB3, ESRRA, FASLG, FGF8, FSTL1, HGF, HPX, ICAM2, IFNB1, IL17B, IL17C, IL17D, IL17F, IL24, IL26, ITLN1, LIF, MFGE8, MMP20, NBL1, NPY, NRG2, PGF, SPARC, TF, TFPI, VWF
	Extracellular space	32: ANGPTL2, APLN, CCK, CCL15, CCL7, CER1, DCN, DLK1, ERBB3, FASLG, FGF8, FSTL1, HGF, HPX, IFNB1, IL17B, IL17C, IL17D, IL17F, IL24, IL26, LIF, MFGE8, MMP20, NBL1, NPY, NRG2, PGF, SPARC, TF, TFPI, TIMP2
	Plasma membrane part	18: CCBP2, CCR1, CCR2, CSK, CTNNB1, DLK1, EDA, ERBB3, ERBB4, FGF8, HTRA2, ICAM2, ITLN1, LZTS1, MFGE8, MFRP, MPL, TF
	Cell surface	10: CCR1, DLK1, FASLG, FGF8, MFGE8, MPL, SPARC, TF, TFPI, TIMP2
	Side of membrane	7: CCR1, CSK, DLK1, FASLG, FGF8, HTRA2, MFGE8
	Cytoplasmic membrane-bounded vesicle lumen	6: FASLG, HGF, HPX, SPARC, TF, VWF
	Secretory granule lumen	4: HGF, SPARC, TF, VWF
Upregulated DEPs	Extracellular region	37: APOE, CCL1, CDH13, CHRDL2, CNTF, CRP, CTSB, DAND5, DLL1, EPO, FGF18, FGL1, FST, GAL, GAST, GCG, HCRT, IL31, IL34, ITGA2B, KLK14, LCN2, LGMN, MFNG, MMP9, NTF4, OLR1, PAPPA, PGA5, PGLYRP1, RARRES2, SAA1, SCARB2, SCG3, SERPINA9, SLC2A5, TFRC
	Extracellular region part	30: APOE, CCL1, CDH13, CHRDL2, CNTF, CRP, CTSB, EPO, FGF18, FGL1, GAL, GCG, IL31, IL34, ITGA2B, KITLG, KLK14, LCN2, LGMN, MFNG, MMP9, OLR1, PGA5, PGLYRP1, RARRES2, SAA1, SCARB2, SERPINA9, SLC2A5, TFRC
	Extracellular space	24: APOE, CCL1, CDH13, CHRDL2, CNTF, CRP, CTSB, EPO, FGF18, FGL1, GAL, GCG, IGF2, IL31, IL34, ITGA2B, KITLG, KLK14, LCN2, MFNG, MMP9, SAA1, SERPINA9, TFRC
	Vesicle	23: APOE, CDH13, CRP, CTSB, DLL1, FGL1, GAL, GCG, IGF2, ITGA2B, KLK14, LCN2, LGMN, MMP9, OLR1, PGA5, PGLYRP1, RARRES2, SAA1, SCARB2, SCG3, SLC2A5, TFRC
	Membrane-bounded vesicle	22: APOE, CDH13, CRP, CTSB, FGL1, GAL, GCG, IGF2, ITGA2B, KLK14, LCN2, LGMN, MMP9, OLR1, PGA5, PGLYRP1, RARRES2, SAA1, SCARB2, SCG3, SLC2A5, TFRC
	Extracellular exosome	19: APOE, CDH13, CRP, CTSB, FGL1, IGF2, ITGA2B, KLK14, LCN2, LGMN, MMP9, OLR1, PGA5, PGLYRP1, RARRES2, SAA1, SCARB2, SLC2A5, TFRC
	Cytoplasmic vesicle	10: APOE, CTSB, DLL1, GAL, GCG, IGF2, ITGA2B, SAA1, SCG3, TFRC
	Secretory granule	6: GAL, GCG, HCRT, IGF2, ITGA2B, SCG3
	Side of membrane	6: CCR7, CDH13, CTSB, ITGA2B, NF1, TFRC
	Cytoplasmic membrane-bounded vesicle lumen	5: APOE, GCG, IGF2, SAA1, SCG3
	External side of plasma membrane	5: CCR7, CDH13, CTSB, ITGA2B, TFRC


**Table 3C:** Molecular function of differentially expressed proteins (DEPs).

DEPs	Item description	Protein count and matching proteins
Downregulated DEPs	Protein binding	39: ADAM11, ANGPTL2, APLN, CCK, CCL15, CCL7, CCR1, CCR2, CDH5, CER1, CSK, CTNNB1, EDA, ERBB3, ERBB4, ESRRA, FASLG, FGF8, HGF, HTRA2, ICAM2, IFNB1, IL17B, IL17C, IL17F, IL24, IL26, LIF, NBL1, NGF, NPY, NRG2, NRTN, NTF3, PGF, SPARC, TF, TIMP2, VWF
	Receptor binding	31: ADAM11, ANGPTL2, APLN, CCK, CCL15, CCL7, CCR2, CDH5, CSK, CTNNB1, EDA, ERBB4, FASLG, FGF8, HGF, ICAM2, IFNB1, IL17B, IL17C, IL17F, IL24, IL26, LIF, NGF, NPY, NRG2, NRTN, NTF3, PGF, TF, VWF
	Cytokine activity	9: CCL15, CCL7, IFNB1, IL17B, IL17C, IL17F, IL24, IL26, LIF
	Cytokine receptor binding	7: CCL15, CCL7, CCR2, IFNB1, LIF, NGF, NTF3
	Growth factor activity	6: FGF8, HGF, LIF, NGF, NTF3, PGF
	Chemoattractant activity	4: CCL15, FGF8, HGF, NTF3
	Cytokine binding	4: CCR1, CER1, IL17F, NBL1
	C-C chemokine receptor activity	3: CCBP2, CCR1, CCR2
	Morphogen activity	2: CER1, NBL1
	BMP binding	2: CER1, NBL1
Upregulated DEPs	Protein binding	27: ADAM17, APOE, CCL1, CCR7, CDH13, CNTF, CRP, CTSB, DLL1, FGF18, FST, GAL, GAST, GCG, HSPB6, IGF2, IL34, ITGA2B, KITLG, MMP9, NTF4, RARRES2, SAA1, SCARB2, SMAD4, SMAD5, TFRC
	Receptor binding	16: ADAM17, APOE, CCL1, CNTF, CRP, DLL1, FGF18, GAL, GAST, GCG, IGF2, IL34, KITLG, NTF4, RARRES2, SAA1
	Endopeptidase activity	7: ADAM17, CTSB, KLK14, LGMN, MMP9, PAPPA, PGA5
	Growth factor activity	4: CNTF, FGF18, IGF2, NTF4
	Lipoprotein particle binding	3: APOE, CDH13, CRP
	Interleukin-6 receptor binding	2: ADAM17, CNTF


### PPI Network Analysis of the DEPs

**FIGURE 2:**
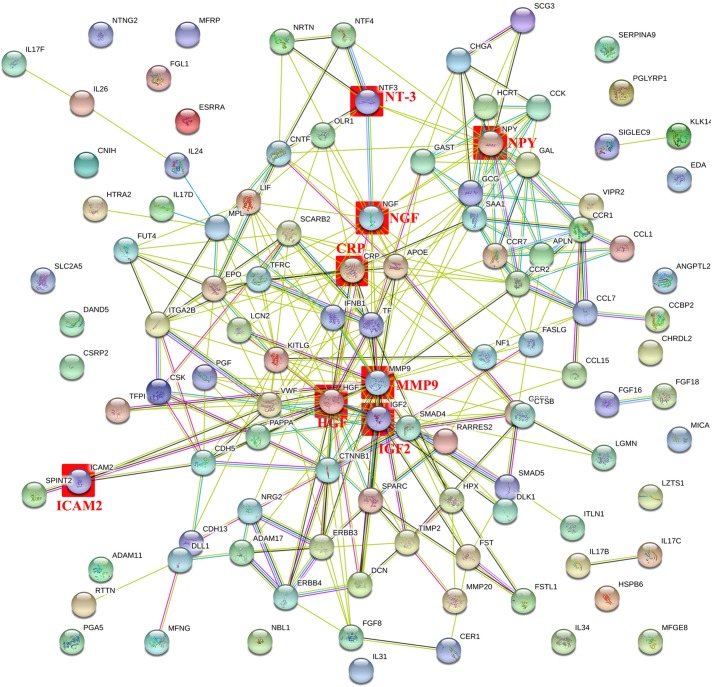
Intermolecular interactions of differentially expressed proteins (DEPs). The intermolecular interactions of all DEPs are shown in this figure. The lines reflect interaction relationship of DEPs. Network edges: line color indicates the type of interaction evidence from the interaction sources, line thickness indicates the strength of data support, and line shape indicates the predicted mode of action. The interaction sources: text mining, experiments, databases, co-expression, neighborhood, gene fusion, and co-occurrence. The minimum required interaction score was medium confidence 0.400. The max number of interactors to show: none or query proteins only in first shell and none in second shell. The network display mode: interactive svg (a scalable vector graphic). The set organism: homo sapiens. Colored nodes: query proteins and first shell of interactors, white nodes: second shell of interactors, empty nodes: proteins of unknown 3D structure, filled nodes: proteins with known or predicted 3D structure.

The PPI network with obvious interaction relationship for all DEPs included 109 nodes and 293 edges, in which the network of downregulated proteins included 58 nodes and 82 edges, and the PPI network of upregulated proteins composed of 51 nodes and 74 edges (Figure 2). Additionally, the top five pairs of all DEPs with the greatest combined score were TF-TFRC (0.999), CCK-GAST (0.993), NRG2-ERBB3 (0.991), GCG-GAST (0.99), and CTNNB1-CDH5 (0.988) (Figure 2 and Supplementary Data Sheet S6). Moreover, the top 10 proteins with the max number of interactors in the PPI network of all DEPs were MMP9 (30), HGF (25), CRP (19), NGF (18), NPY (17), VWF (17), IGF-2 (15), KITLG (15), TF (15), and EPO (13, the same as GCG and SAA1) (Figure 2 and Supplementary Data Sheet S6). Meanwhile, the top five pairs with the greatest combined score were NRG2-ERBB3 (0.991), CTNNB1-CDH5 (0.988), NRG2-ERBB4 (0.984), CCL7-CCR2 (0.978), CCL7-CCR1 (0.959) in the downregulated proteins, and GCG-GAST (0.99), EPO-KITLG (0.982), SMAD5-SMAD4 (0.968), GCG-VIPR2 (0.951), IGF2-PAPPA (0.948) in upregulated proteins (Figure 2 and Supplementary Data Sheet S6). Then the top five proteins with the max number of interactors were HGF (15), NGF (10), VWF (8), CTNNB1 (7), NPY (7) in the PPI network of downregulated proteins, and CRP (12), MMP9 (11), APOE (8), GCG (8), SAA1 (8) in the PPI network of upregulated proteins (Figure 2 and Supplementary Data Sheet S6). In summary, NGF (18), NT-3 (7), IGF-2 (15), HGF (18), NPY (17), CRP (19), MMP-9 (30) and ICAM-2 (2) hold the high number of interactors (shown in the brackets) and might be the core protein in PPI Network of all DEPs. Therefore, they were considered to be meaningful proteins and were for further study. Here values in brackets are the combined score value or the number of interactors.

### Kyoto Encyclopedia of Genes and Genomes (KEGG) Analyses for Differentially Expressed Proteins (DEPs)

To analyze the enriched pathways of DEPs, KEGG analysis was performed. The results showed that the significant enriched pathways of the downregulated DEPs were cytokine–cytokine receptor interaction, PI3K-Akt signaling pathway, proteoglycans in cancer, Rap1 signaling pathway, Ras signaling pathway, and Jak-STAT signaling pathway (Table 4). Meanwhile, the enriched pathways of the upregulated DEPs were showed as follows: pathways in cancer, cytokine–cytokine receptor interaction, hematopoietic cell lineage and Notch signaling pathway (Table 4). Among these pathways, they were either relevant to growth or inflammation.

**Table 4:** KEGG pathways classification of differentially expressed proteins.

DEPs	Pathway description	Protein count	Matching proteins
Upregulated DEPs	Cytokine–cytokine receptor interaction	11	CCL7, CCR1, CCR2, EDA, FASLG, HGF, IFNB1, IL17B, IL24, IL26, MPL
	PI3K-Akt signaling pathway	7	FASLG, FGF8, HGF, IFNB1, NGF, PGF, VWF
	Proteoglycans in cancer	6	CTNNB1, DCN, ERBB4, FASLG, FGF8, HGF
	Rap1 signaling pathway	5	CTNNB1, FGF8, HGF, NGF, PGF
	Ras signaling pathway	5	FASLG, FGF8, HGF, NGF, PGF
	Jak-STAT signaling pathway	4	IFNB1, IL24, IL26, MPL
Downregulated DEPs	Pathways in cancer	6	FGF16, FGF18, ITGA2B, KITLG, MMP9, SMAD4
	Cytokine–cytokine receptor interaction	5	CCL1, CCR7, CNTF, EPO, KITLG
	Hematopoietic cell lineage	4	EPO, ITGA2B, KITLG, TFRC
	Notch signaling pathway	3	ADAM17, DLL1, MFNG


### Changes of Expression of Some Growth Factors and Inflammation-Related Factors After TBI Injury

**FIGURE 3:**
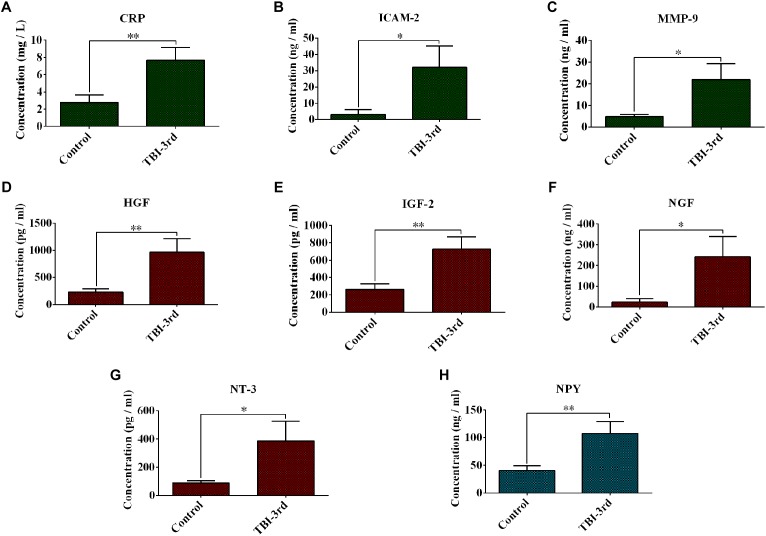
Changes of expression of some growth factors and inflammation-related factors in the serum. **(A–H)** Separately showed the serum concentration of CRP, ICAM-2, MMP-9, HGF, IGF-2, NGF, NT-3, and NPY in control group and TBI-third group. The data was represent as mean ± SEM. *^∗^*TBI vs. Control, ^∗^*P* < 0.05, ^∗∗^*P* < 0.01; ^∗∗∗^*P* < 0.001.

Because of the high number of interactors of NGF, NT-3, IGF-2, HGF, NPY, CRP, MMP-9, and ICAM-2 in PPI Network, we think they may play a pivotal role in traumatic brain injury. In our study, therefore, their expression in serum was verified by ELISA. 39 sera from 19 patients in control group and 20 patients in TBI-third group were detected. Compared with control group, CRP, ICAM-2, and MMP-9 from innate immune system were significantly increased in TBI-third group, and the *P*-values were 0.008, 0.045, 0.03, respectively (Figure 3A–C). In addition, HGF, IGF-2, NGF, and NT-3 with growth factor activity were also up-regulated in TBI-third group in comparison with control group, and the difference was statistically significant, and the *P*-value were 0.038, 0.042, 0.006, 0.009, respectively (Figure 3D–G). Furthermore, NPY with neuropeptide hormone activity was significantly more in TBI-third group than that in control group (*P* = 0.009) (Figure 3H). Moreover, the mean ± SEM value and the 95% CI of the concentration of the eight proteins in the serum of both groups were shown Table 5.

**Table 5:** The mean ± SEM and 95% confidence interval (CI) of the concentration of NGF, NT-3, IGF-2, HGF, CRP, ICAM-2, MMP-9, and NPY in the serum.

Proteins	Group	Mean ± SEM	95% CI
NGF	Control	(23.80 ± 16.32) *ng/ml*	(0˜58.09) ng/ml
	TBI-third	(242.16 ± 96.96) *ng/ml*	(39.21˜445.10) ng/ml
NT-3	Control	(76.46 ± 12.10) *pg/ml*	(50.93˜101.99) pg/ml
	TBI-third	(384.84 ± 140.90) *pg/ml*	(89.92˜679.75) pg/ml
IGF2	Control	(262.07 ± 63.68) *pg/ml*	(127.72˜396.41) pg/ml
	TBI-third	(727.37 ± 141.57) *pg/ml*	(431.06˜1023.67) pg/ml
HGF	Control	(229.40 ± 59.76) *pg/ml*	(103.86˜354.95) pg/ml
	TBI-third	(965.60 ± 251.10) *pg/ml*	(441.81˜1489.39) pg/ml
CRP	Control	(2.77 ± 0.89) *mg/L*	(0.90˜4.64) mg/L
	TBI-third	(7.65 ± 1.51) *mg/L*	(4.49˜10.81) mg/L
ICAM2	Control	(3.09 ± 3.04) *ng/ml*	(0˜9.05) ng/ml
	TBI-third	(32.05 ± 13.22) *ng/ml*	(4.37˜59.72) ng/ml
MMP9	Control	(4.79 ± 1.00) *ng/ml*	(2.68˜6.90) ng/ml
	TBI-third	(21.98 ± 7.28) *ng/ml*	(6.74˜37.21) ng/ml
NPY	Control	(40.84 ± 8.71) *ng/ml*	(22.53˜59.15) ng/ml
	TBI-third	(107.36 ± 21.88) *ng/ml*	(61.55˜153.16) ng/ml


## Discussion

In this study, the serum samples of TBI patients and healthy volunteers were subjected to protein microarray analysis and it was found that there were significant changes in many proteins, especially growth factors and inflammatory factors in the serum after TBI injury compared with control group. As everyone knows, clinical data is often influenced by many factors. In order to ensure the scientificalness of the study, the mean age and age distribution of patients in TBI-third group were the same as those in the control group. Moreover, the major indicators such as liver function, renal function and blood lipids in both groups were within normal reference range, which indicated that the liver and renal function of all cases are normal. Furthermore, all volunteers in the control group must have no other organic-related diseases, and all patients in the TBI-third group have mild-moderate damage with 11–15 points of GCS but without other organic diseases except for TBI injury. Therefore, the baseline data of both groups were almost consistent.

At the same time, in this study, the degree of traumatic brain injury was determined by CT performance and GCS score. Because of the rapid acquisition of CT images and independent changes in the patient’s consciousness, routine CT examinations are known as the “gold standard” for the diagnosis of acute brain injury (Toyama et al., 2005). However, CT showed poor brain parenchymal lesions, and it only had a good effect on diagnosing large-area hemorrhagic changes, large-area brain contusion and other relatively wide-ranging lesions. There is a big limitation on the diagnosis of microbleeds in brain tissue, so that the severity of traumatic brain injury was often missed or judged too lightly (Brophy et al., 2011). Moreover, in the acute phase of TBI, the patients may be in a coma or critically ill and then unable to complete the large-scale tests. Therefore, CT performance combined with the clinical behavioral observations to assess the degree of damage is more reliable. The most common clinical behavioral observation is the GCS score, which scored according to coma severity (Becker et al., 2018). In this study, CT showed that the injured sites of the patients with TBI were in forehead, partes temporalis, facies parietalis and occiput, and 85% of the patients had the intracerebral, epidural or subdural hematoma. In addition, the GCS scores of these patients were 11–15 points, that is, mild and moderate coma. Therefore, the serum samples from TBI patients used for in this study also were credible.

Through bioinformatics analysis, we found the biological process of DEPs, the most of which happened in the extracellular region, extracellular region part and extracellular space, was involved in positive/negative regulation of cellular process, signaling and signal transduction, cell communication, response to stimulus, immune system process, multicellular organismal development, metabolic process, and biological process. Moreover, the molecular functions of them were involved in receptor binding, protein binding, cytokine activity, growth factor activity, chemoattractant activity, cytokine receptor binding, C-C chemokine receptor activity, cytokine binding, morphogen activity, BMP binding, and interleukin-6 receptor binding. Additionally, the most significant pathways were enriched in cytokine–cytokine receptor interaction and PI3K-Akt signaling pathway among downregulated proteins, and pathways in cancer and cytokine–cytokine receptor interaction among upregulated proteins. To sum up, these DEPs were much enriched in the biological processes and pathways related with growth and inflammation. As noted above, the secondary injury of traumatic brain injury involves a series of cellular processes (Xiong et al., 1999; Park et al., 2008), which are the result of the involvement of these DEPs. By binding with other molecules (e.g., protein, receptor, and cytokine) and the activity of growth factors and inflammatory factors, they are involved in positively or negatively regulating cellular process, signal transduction, cell communication, response to stimulus, immune system process and multicellular organismal development, etc. Then, in turn, they further promote the damage repair, or suppress or even worsen damage.

The role of growth factors and inflammatory factors in the body is mutual resistance. Growth factors can promote the recovery of neurologic damage. In contrast, inflammatory factors destroy the repair. If the growth factors and anti-inflammatory factors predominate, the prognosis of TBI patients is better; however, if the injury factors such as inflammation prevail, the prognosis will be worse. After TBI, both physical and chemical factors activate the innate immune system to cause a cascade of inflammatory reactions at the site of the injury. Inflammatory factors accumulate around the injury and diffuse into the cerebrospinal fluid and the blood, which is inextricably linked to the patient’s neurological outcome. In our study, WBC and NEUT in TBI-third group were increased and more than the upper limit of normal reference range, which demonstrated that acute inflammatory reaction existed. What’s more, inflammation-related factors including CRP, ICAM-2, and MMP-9 were also elevated, suggesting that after traumatic brain injury, the innate immune system was activated, then the inflammatory factors damaged the blood–brain barrier and were released into the blood. Previous studies also reported similar results. After TBI, neurological function of rats was impaired, while immediately administrating with propofol for 2 h after TBI could effectively improve neurological function (Liu et al., 2016). Simultaneously, the expressions of IL-1β, IL-6, and TNF-α within 1 week after operation were significantly augmented in the injured cortex, but markedly decreased after propofol treatment (Liu et al., 2016). In addition, after TBI injury, some interventions, such as administrated with breviscapine via the right lateral ventricle or co-transplanting NSCs with olfactory ensheathing cells (OECs) into the area surrounding the injury site, could reduce interleukin-6 expression, and significantly improved neurobehavioral dysfunction in the short-term (Liu et al., 2014; Jiang et al., 2017).

On the other hand, however, it has also been reported that growth factors were involved in nerve repair. In traumatic brain injury, neurological function improvement was associated with the upregulated BDNF expression, while the blockade of BDNF exacerbated neurological function deficits (Xiong et al., 2017). Similarly, after spinal cord injury (SCI), the level of BDNF also gradually increased with partial functional restoration, and BDNF overexpressing facilitated the recovery of locomotor function, while BDNF knockdown wound led to opposite outcome (Gao et al., 2012; Chen et al., 2016). Moreover, GDNF and CNTF played an essential role in neuroplasticity after TBI and the SCI, which could downregulate the expression of BAX and BAD signaling to suppress apoptosis, therefore, to improve neurological function and survival (Qin et al., 2016; Qiu and Wang, 2016; Feng et al., 2017). Furthermore, the upregulation of NGF and NT-3 in neurons also could enhance neurons’ survival and neurite outgrowth, as well as promoted motor and sensory function recovery in hind limbs after SCI (Yang et al., 2009; Wang et al., 2016; Xiong et al., 2016). At present, we investigated the factors associated with growth and nerve repair by ELISA, including NGF, NT-3, HGF, IGF-2, NPY in serum, and they were found to be elevated in TBI-third group compared to the control group, which pointed out that the protective factors were playing a neuroprotective role after TBI injury.

Therefore, after TBI injury, it is a trend to inhibit its damage factors, especially the progression of inflammatory factors, and promote its protective factors, especially the growth factors and neuropeptides, which will contribute to clinical diagnosis and targeted therapies of TBI in translational medicine and clinical practice.

## Conclusion

In this study, we reported the protein expressional profiles in serum from TBI patients, in which the network among inflammatory factors and growth factors may play a crucial role in TBI damage and repair, which, consequently, may contribute to diagnosis and treatment in future translational medicine and clinical practice.

## Author Contributions

T-HW was participated in the guidance and design of the study and the revision of the paper. X-YH and Q-QD were responsible for the design of the study, the manuscript writing and revision, ELISA, statistical analysis, and data description. FW was performed the data ordering and analysis, supervised the experiments, and revised the manuscript. NZ, S-JF, and Y-KL were performed the sample collection, data analysis, and revised the manuscript. All authors have read and approved the final version of the manuscript.

## Conflict of Interest Statement

The authors declare that the research was conducted in the absence of any commercial or financial relationships that could be construed as a potential conflict of interest.

## Footnotes

1 https://string-db.org/cgi/input.pl

## Supplementary Material

Data Sheet S1 Basic information of patients for protein chips.

Data Sheet S2 Human protein chip (1000 proteins included).

Data Sheet S3 The Differential Expressed Proteins (DEPs).

Data Sheet S4 The GO and Pathway analysis of downregulated DEPs.

Data Sheet S5 The GO and Pathway analysis of upregulated DEPs.

Data Sheet S6 The interactions in tabular form.

## Abbreviations

BAD: Bcl-2 associated death promoter
BAX: Bcl-2 associated X protein
BDNF: brain-derived neurotrophic factor
BMPs: bone morphogenetic proteins
CNTF: ciliary neurotrophic factor
CRP: C-reactive protein
CT: computed tomography
GDNF: glial cell-derived neurotrophic factor
HGF: hepatocyte growth factor
HRP: horse radish peroxidase
ICAM2: intercellular adhesion molecule 2
IGF2: insulin like growth factor 2
MMP9: matrix metallopeptidase 9
NGF: nerve growth factor
NPY: neuropeptide Y
NT-3: neurotrophin 3
TGF-β: transforming growth factor β
