# Supplementary material for: Protein Network Analysis of the Serum and Their Functional Implication in Patients Subjected to Traumatic Brain Injury
Source: Front Neurosci. 2019 Jan 31;12:1049. doi: 10.3389/fnins.2018.01049 (PMC6365836; doi:10.3389/fnins.2018.01049)
Supplement: Data Sheet S1 — Basic information of patients for protein chips. [file Table_1.DOCX]

**Supplementary data 1. Basic information of patients for protein chips.**

| Group | Control group | TBI-3rd group |
| --- | --- | --- |
| Gender | Female: one, Male: Two | Female: one, Male: Two |
| Age (Year) | 47.67±24.11 | 46.67±23.07 |
| Cause of injury | None | Trauma |
| GCS scores | 15 | 13.33±1.15 |
| Hematoma | None | Intracerebral |
| Bleeding volume (ml) | None | 7.30±3.20 |
| Treatment | None | Expectant Treatment |
