# Supplementary material for: Protein Network Analysis of the Serum and Their Functional Implication in Patients Subjected to Traumatic Brain Injury
Source: Front Neurosci. 2019 Jan 31;12:1049. doi: 10.3389/fnins.2018.01049 (PMC6365836; doi:10.3389/fnins.2018.01049)
Supplement: Data Sheet S2 — Human protein chip (1000 proteins included). [file Table_2.doc]

The RayBio® Biotin label-based human antibody arrays used in this study could detect the expression of 1000 human protein, including cytokines, chemokines, adipokines, growth factors, proteases, soluble antibodies, adhesion factors and other proteins. They are shown as follows.

11b-HSD1, 2B4, 4-1BB, 6Ckine, A1BG, A2M, ABL1, ACE, ACE-2, ACK1, ACPP, ACTH, Activin A, Activin B, Activin C, Activin RIA / ALK-2, Activin RIB / ALK-4, Activin RII A/B, Activin RIIA, ADAM-9, ADAMTS-1, ADAMTS-10, ADAMTS-13, ADAMTS-15, ADAMTS-17, ADAMTS-18, ADAMTS-19, ADAMTS-4 , ADAMTS-5, ADAMTS-L2, Adiponectin / Acrp30, Adipsin, Afamin, AFP, AgRP, ALBUMIN, ALCAM, Aldolase A, Aldolase B, Aldolase C, ALK, Alpha 1 AG, Alpha 1 Microglobulin, Alpha Lactalbumin, ALPP, AMICA, AMPKa1, Amylin, Angiogenin, Angiopoietin-1, Angiopoietin-2, Angiopoietin-4, Angiopoietin-like 1, Angiopoietin-like 2, Angiopoietin-like Factor, Angiostatin, ANGPTL3, ANGPTL4, Annexin A7, APC, APCS, Apelin, Apex1, APJ, APN, ApoA1, ApoA2, ApoA4, ApoB, ApoB100, ApoC1, ApoC2, ApoC3, ApoD, ApoE, ApoE3, ApoH, ApoM, APP, APRIL, AR (Amphiregulin), Artemin, ASPH, Attractin, Axl, B3GNT1, B7-1 /CD80, BACE-1, BAF57, BAFF, BAFF R / TNFRSF13C, BAI-1, bax, BCAM, BCMA / TNFRSF17, BD-1, BDNF, Beta 2M, Beta Defensin 4, Beta IG-H3, beta-Catenin, beta-NGF, Biglycan, BIK, BLAME, BLC / BCA-1 / CXCL13, BMP-15, BMP-2, BMP-3, BMP-3b / GDF-10, BMP-4, BMP-5, BMP-6, BMP-7, BMP-8, BMP-9, BMPR-IA / ALK-3, BMPR-IB / ALK-6, BMPR-II, BMX, BNIP2, BNP, BTC, Btk, C2, C3a, C5/C5a, C7, C8B, C9, CA125, CA15-3, CA19-9, CA9, Cadherin-13, Calbindin, Calbindin D, Calcitonin, Calreticulin, Calsyntenin-1, Cardiotrophin-1 / CT-1, CART, Caspase-3, Caspase-8, Cathepsin B, Cathepsin D, Cathepsin L, Cathepsin S, CBP, CCK, CCL14 / HCC-1 / HCC-3, CCL28 / VIC, CCR1, CCR2, CCR3, CCR4, CCR5, CCR6, CCR7, CCR8, CCR9, CD 163, CD14, CD200, CD23, CD24, CD27 / TNFRSF7, CD30 / TNFRSF8, CD30 Ligand / TNFSF8, CD36, CD38, CD40 / TNFRSF5, CD40 Ligand / TNFSF5 /CD154, CD44, CD45, CD46, CD47, CD55, CD59, CD61, CD71, CD74, CD79 alpha, CD90, CD97, CEA, CEACAM-1, Cerberus 1, Ceruloplasmin, CFHR2, Chem R23, Chemerin, CHI3L1, Chordin-Like 1, Chordin-Like 2, Chromogranin A, Chymase, cIAP-2, Ck beta 8-1, CK-MB, Claudin-3, Claudin-4, CLC, CLEC3B, Clusterin, CNDP1, CNTF R alpha, CNTF, Coagulation Factor III / Tissue Factor, Coagulation Factor X/Xa, COCO, Complement factor H, Contactin-1, Contactin-2, Corticosteroid-binding globulin, COX-2, C-peptide, CPN2, Creatinine, CRIM 1, Cripto-1, CRP, CRTAM, CRTH-2, Cryptic, CSH1, Csk, CTACK / CCL27, CTGF / CCN2, CTLA-4 /CD152, cTnT / Troponin T, CutA, CV-2 / Crossveinless-2, CXCL14 / BRAK, CXCL16, CXCR1 / IL-8 RA, CXCR2 / IL-8 RB, CXCR3, CXCR4 (fusin), CXCR5 /BLR-1, CXCR6, Cyclin D1, Cystatin A, Cystatin B, Cystatin C, Cytochrome C, Cytokeratin 8, Cytokeratin18, Cytokeratin19, D6, DAN, DANCE, DBI, DCBLD2, DcR3 / TNFRSF6B, D-Dimer, Decorin, DEFA1/3, Defensin, Desmin, Dkk-1, Dkk-3, Dkk-4, DLL1, DLL4, DMP-1, DPPIV, DR3 / TNFRSF25, DR6 / TNFRSF21, Dtk, E-Cadherin, EDA-A2, EDAR, EDG-1, EGF, EGF R / ErbB1, EG-VEGF / PK1, EMAP-II, ENA-78, Endocan, Endoglin / CD105, Endorphin Beta, Endostatin, Endothelin, Endothelin Receptor A, Enolase 2, ENPP2, EN-RAGE, Eotaxin / CCL11, Eotaxin-2 / MPIF-2, Eotaxin-3 / CCL26, EpCAM, EphA1, EphA2, EphA3, EphA4, EphA5, EphA6, EphA7, EphA8, EphB1, EphB2, EphB3, EphB4, EphB6, Epiregulin, ErbB2, ErbB3, ErbB4, ERRa, Erythropoietin R, Erythropoietin, ESAM, E-Selectin, EV15L, EXTL2, FABP1, FABP2, FABP3, FABP4, Factor XIII A, Factor XIII B, FADD, FAK, FAM3B, FAP, Fas / TNFRSF6, Fas Ligand, Fc RIIB/C, Fen 1, FER, Ferritin, Fetuin A, Fetuin B, FGF Basic, FGF R3, FGF R4, FGF R5, FGF-10 / KGF-2, FGF-11, FGF-12, FGF-13 1B, FGF-16, FGF-17, FGF-18, FGF-19, FGF-20, FGF-21, FGF-23, FGF-4, FGF-5, FGF-6, FGF-7 / KGF, FGF-8, FGF-9, FGF-BP, FGFR1, FGFR1 alpha, FGFR2, Fibrinogen, Fibrinopeptide A, Fibronectin, Ficolin-3, FIH, FLRG, Flt-3 Ligand, Follistatin, Follistatin-like 1, FOLR1, FOXN3, FoxO1, FoxP3, Fractalkine, Frizzled-1, Frizzled-3, Frizzled-4, Frizzled-5, Frizzled-6, Frizzled-7, FRK, FSH, Furin, Fyn, GADD45A, Galanin, Galectin-1, Galectin-3, Galectin-3BP, Galectin-7, gamma-Thrombin, Gas1, GASP-1 / WFIKKNRP, GASP-2 / WFIKKN, Gastrin, GATA-3, GATA-4, GCP-2 / CXCL6, GCSF, G-CSF R / CD 114, GDF1, GDF11, GDF-15, GDF3, GDF5, GDF8, GDF9, GDNF, Gelsolin, GFR alpha-1, GFR alpha-2, GFR alpha-3, GFR alpha-4, Ghrelin, GITR / TNFRF18, GITR Ligand / TNFSF18, GLO-1, GLP-1, Glucagon, Glut1, Glut2, Glut3, Glut5, Glypican 3, Glypican 5, GM-CSF, GM-CSF R alpha, GMNN, GPBB, GPI, GPR-39, GPX1, GPX3, Granzyme A, Grb2, GREMLIN, GRO, GRO-a, Growth Hormone (GH), Growth Hormone R (GHR), GRP, GRP75, GRP78, GSR, GST, HADHA, HAI-1, HAI-2, Haptoglobin, HB-EGF, HCC-4 / CCL16, hCG alpha, hCGb, Hck, HCR / CRAM-A/B, HE4, Hemopexin, Hepassocin, Hepcidin, HGF, HGFR, HOXA10, HRG-alpha, HRG-beta 1, HSP10, HSP20, HSP27, HSP32, HSP40, HSP60, HSP70, HSP90, HSPA8, HTRA2, HVEM / TNFRSF14, I-309, IBSP, ICAM-1, ICAM-2, ICAM-3 (CD50), ICAM-5, IFN-alpha / beta R1, IFN-alpha / beta R2, IFN-beta, IFN-gamma, IFN-gamma R1, IGF2BP1, IGFBP-1, IGFBP-2, IGFBP-3, IGFBP-4, IGFBP-5, IGFBP-6, IGFBP-rp1 / IGFBP-7, IGF-I, IGF-I SR, IGF-II, IGF-II R, IL-1 alpha, IL-1 beta, IL-1 F10 / IL-1HY2, IL-1 F5 / FIL1delta, IL-1 F6 / FIL1 epsilon, IL-1 F7 / FIL1 zeta, IL-1 F8 / FIL1 eta, IL-1 F9 / IL-1 H1, IL-1 R3 / IL-1 R AcP, IL-1 R4 /ST2, IL-1 R6 / IL-1 Rrp2, IL-1 R8, IL-1 R9, IL-1 ra, IL-1 sRI, IL-1 sRII, IL-10, IL-10 R alpha, IL-10 R beta, IL-11, IL-12 p40, IL-12 p70, IL-12 R beta 1, IL-12 R beta 2, IL-13, IL-13 R alpha 1, IL-13 R alpha 2, IL-15, IL-15 R alpha, IL-16, IL-17, IL-17B, IL-17B R, IL-17C, IL-17D, IL-17E, IL-17F, IL-17R, IL-17RC, IL-17RD, IL-18 BPa, IL-18 R alpha /IL-1 R5, IL-18 R beta /AcPL, IL-19, IL-2, IL-2 R alpha, IL-2 R beta /CD122, IL-2 R gamma, IL-20, IL-20 R alpha, IL-20 R beta, IL-21, IL-21 R, IL-22, IL-22 BP, IL-22 R, IL-23, IL-23 R, IL-23p19, IL-24, IL-26, IL-27, IL-28A, IL-29, IL-3, IL-3 R alpha, IL-31, IL-31 RA, IL-33, IL-34, IL36RN, IL-4, IL-4 R, IL-5, IL-5 R alpha, IL-6, IL-6 R, IL-7, IL-7 R alpha, IL-8, IL-9 , INSL3, INSRR, Insulin, Insulin R, Insulysin / IDE, Integrin alpha V, IP-10, I-TAC / CXCL11, Itk, ITM2B, Kallikrein 10, Kallikrein 11, Kallikrein 14, Kallikrein 2, Kallikrein 5, Kallikrein 6, Kallikrein 7, Kallikrein 8, KCC3, KCTD10, KIF3B, Kininostatin / kininogen, KLF4, Kremen-1, Kremen-2, LAG-3, Latent TGF-beta bp1, Layilin, LBP, Lck, LDL R, LECT2, Lefty - A, Legumain, Leptin (OB), Leptin R, LFA-1 alpha, LH, LIF R alpha, LIF, LIGHT / TNFSF14, LIMPII, LIN41, Lipocalin-1, Livin, LOX-1, LPS, LRG1, LRP-1, LRP-6, L-Selectin (CD62L), LTF, LTK, Luciferase, Lumican, Lymphotactin / XCL1, Lymphotoxin beta / TNFSF3, Lymphotoxin beta R / TNFRSF3, Lyn, LYRIC, LYVE-1, LZTS1, MAC-1, Mammaglobin A, Marapsin, MATK, MBL, MBL-2, MCP-1, MCP-2, MCP-3, MCP-4 / CCL13, M-CSF, M-CSF R, MDC, Mer, Mesothelin, MFG-E8, MFRP, MICB, Midkine, MIF, MIG, MINA, MIP 2, MIP-1a, MIP-1b, MIP-1d, MIP-3 alpha, MIP-3 beta, MMP-1, MMP-10, MMP-11 /Stromelysin-3, MMP-12, MMP-13, MMP-14, MMP-15, MMP-16 / MT3-MMP, MMP-19, MMP-2, MMP-20, MMP-24 / MT5-MMP, MMP-25 / MT6-MMP, MMP-3, MMP-7, MMP-8, MMP-9, MSHa, MSP alpha Chain, MSP beta-chain, MTUS1, Musk, Myoglobin, NAIP, Nanog, NAP-2, NCAM-1 / CD56, NELL2, NEP, Nesfatin, Nestin, NET1, Netrin G2, Netrin-4, Neuritin, NeuroD1, Neurokinin-A, Neuropeptide Y, Neuropilin-2, Neurturin, NF1, NGF R, NM23-H1/H2, Notch-1, NOV / CCN3, NPTX1, NPTXR, NR3C3, NRG1 Isoform GGF2, NRG1-alpha / HRG1-alpha, NRG1-beta1 / HRG1-beta1, NRG2, NRG3, NT-3, NT-4, Ntn1, OCT3/4, Omentin, Orexin A, Orexin B, OSM, Osteoactivin / GPNMB, Osteocalcin, Osteocrin, Osteopontin, Osteoprotegerin / TNFRSF11B, OX40, OX40 Ligand / TNFSF4, p21, p27, p53, PAI-1, PAK7, Pancreastatin, Pancreatic Polypeptide, Pappalysin-1, PARC / CCL18, PARK7, P-Cadherin, PCAF, PD-1, PD-ECGF, PDGF R alpha, PDGF R beta, PDGF-AA, PDGF-AB, PDGF-BB, PDGF-C, PDGF-D, PDX-1, PECAM-1 /CD31, PEDF , Pentraxin3 / TSG-14, PEPSINOGEN I, PEPSINOGEN II, Peroxiredoxin 6 (Prdx6), Persephin, PF4 / CXCL4, PGRP-S, PI 16, PI 3Kinase p85 beta, PIM2, PKM2, Plasminogen, PlGF, PLUNC, Podocalyxin, POMC, PON1, PON2, PPARg2, PPP2R5C, Pref-1, Presenilin 1, Presenilin 2, Pro-BDNF, Procalcitonin, Pro-Cathepsin B, Progesterone, pro-Glucagon, Progranulin, Prohibitin, Prolactin, Pro-MMP-13, Pro-MMP-7, Pro-MMP-9, ProSAAS, Prostasin, Protein p65, PSA-Free, PSA-total, P-selectin, PSP, PTH, PTHLP, PTN, PTPRD, PYK2, PYY, RAGE, RANK / TNFRSF11A, RANTES, Ras, RBP4, RECK, RELM alpha, RELM beta, RELT / TNFRSF19L, Resistin, RET, RIP1, ROBO4, ROCK1, ROCK2, ROR1, ROR2, ROS, RYK, S100 A8/A9, S100A10, S100A4, S100A6, S100A8, S-100b, SAA, SART1, SART3, SCF, SCF R /CD117, SCG3, SDF-1 / CXCL12, Selenoprotein P, SEMA3A, Serotonin, Serpin A1, Serpin A12, Serpin A3, Serpin A4, Serpin A5, Serpin A8, Serpin A9, Serpin B5, Serpin D1, Serpin I1, SERTAD2, sFRP-1, sFRP-3, sFRP-4, sgp130, SHBG, SIGIRR, Siglec-5/CD170, Siglec-9, SLPI, SMAC, Smad 1, Smad 4, Smad 5, Smad 7, Smad 8, SNCG, Soggy-1, Somatotropin, Sonic Hedgehog (Shh N-terminal), SOST, SOX17, SOX2, SPARC, SPARCL1, Spinesin, SPINK1, SRMS, SSEA-1, SSEA-4, SSTR2, SSTR5, Survivin, SYK, Syndecan-1, Syndecan-3, TACE, TACI / TNFRSF13B, TAF4, Tarc, TCCR / WSX-1, Tec, TECK / CCL25, TFF1, TFF3, TFPI, TGF-alpha, TGF-beta 1, TGF-beta 2, TGF-beta 3, TGF-beta 5, TGF-beta RI / ALK-5, TGF-beta RII, TGF-beta RIII, Thrombin, Thrombomodulin, Thrombopoietin (TPO), Thrombospondin-1, Thrombospondin-2, Thrombospondin-4, Thymidine Kinase-1, Thymopoietin, Thyroglobulin, Thyroid Peroxidase (TPX), Tie-1, Tie-2, TIM-1, TIMP-1, TIMP-2, TIMP-3, TIMP-4, TL1A / TNFSF15, TLR1, TLR2, TLR3, TLR4, TMEFF1 / Tomoregulin-1, TMEFF2, TNF RI / TNFRSF1A, TNF RII / TNFRSF1B, TNF-alpha, TNF-beta, TNK1, TOPORS, TPA, TPM1, TRA-1-60, TRA-1-81, TRADD, TRAIL R1 / DR4 / TNFRSF10A, TRAIL R2 / DR5 / TNFRSF10B, TRAIL R3 / TNFRSF10C, TRAIL R4 / TNFRSF10D, TRAIL / TNFSF10, TRANCE, Transferrin, Trappin-2, TREM-1, TRKB, Troponin C, Troponin I, TROY / TNFRSF19, TRPC1, TRPC6, TRPM7, Trypsin 1, TSG-6, TSH, TSLP, TSLP, TWEAK / TNFSF12, TWEAK R / TNFRSF12, TXK, Tyk2, TYRO10, Ubiquitin+1, uPA, uPAR, Uromodulin, Vasopressin, Vasorin, VCAM-1 (CD106), VDUP-1, VE-Cadherin, VEGF, VEGF R1, VEGF R2 (KDR), VEGF R3, VEGF-B, VEGF-C, VEGF-D, VEGI / TNFSF15, VGF, VIP Receptor 2, Visfatin, Vitamin D Receptor, Vitamin D-BP, Vitamin K-dependent protein S, Vitronectin, VWF, WIF-1, Wilms Tumor 1, WISP-1 / CCN4, XEDAR, XIAP, ZAG, ZAP70
